# Improvement in facial seborrheic dermatitis following cervical transcutaneous vagal nerve stimulation

**DOI:** 10.1016/j.jdcr.2025.04.020

**Published:** 2025-04-26

**Authors:** William J. Nahm, Ki-Joong Kim, Eui Namgung, Vincent Falanga, Ryan Chen, David A. Lee, Katrina Schmitt, John Koo

**Affiliations:** a New York University Grossman School of Medicine, New York, New York; b Department of Oriental Medicine, Institute of Bioscience and Integrative Medicine, Daejeon University, Daejeon, South Korea; c Department of Physics, University of California San Diego, La Jolla, California; d Department of Dermatology, Boston University Chobanian and Avedisian School of Medicine, Boston, Massachusetts; e Department of Biochemistry & Cell Biology, Boston University Chobanian and Avedisian School of Medicine, Boston, Massachusetts; f UMass Chan Medical School, Worcester, Massachusetts; g Damascus Dermatology & Skin Surgery Center, Damascus, Maryland; h Baylor Scott and White Health, Dallas, Texas; i Department of Dermatology, University of California San Francisco, San Francisco, California

**Keywords:** headaches, inflammatory skin disease, interferon-γ, seborrheic dermatitis, transcutaneous, vagus nerve, vagal nerve stimulation

## Introduction

The vagus nerve is a critical bidirectional communication pathway between the central nervous system and multiple organs. This mixed nerve facilitates the transmission of both afferent sensory signals and efferent impulses, functioning as a principal conduit for the reciprocal exchange of physiological information.^1^ The innervation provided by the vagus nerve plays a crucial role in maintaining homeostasis through its involvement in autonomic regulation, neuroendocrine function, and immunomodulation. The vagus nerve also plays a role in the inflammatory reflex, a neurophysiological mechanism that modulates innate immunity and regulates inflammatory responses during pathogenic invasion and tissue injury. It is, therefore, a regulator of systemic inflammation.^1^ Invasive vagal nerve stimulation (VNS) has aided patients with Alzheimer’s disease, depression, stroke, tinnitus, and epilepsy.^2^

There have been suggestions that VNS could impact inflammatory skin diseases, such as psoriasis, eczema, and seborrheic dermatitis (SD).^3^ Recently, preclinical studies have demonstrated that invasive vagal nerve stimulation can decrease epidermal hyperplasia and proinflammatory cytokines in murine models of psoriasis and eczema.^4^ Moreover, transcutaneous vagal nerve stimulation (tVNS) has recently been introduced through the cervical and auricular pathways, which are noninvasive, much less expensive, and typically without side effects.^2^ Although VNS has demonstrated efficacy in neurological, inflammatory, gastrointestinal, and arthritic conditions, its role in inflammatory skin diseases remains unexplored. Herein, we present the first case of improvement of SD with tVNS.

## Case report

A 56-year-old male with a history of nonmigraine headaches, post-traumatic stress disorder, prediabetes, musculoskeletal pain disorder, and a long-standing history of SD decided to employ tVNS for his headaches. He procured a cervical tVNS device (radiofrequency transmitter: 2.379 to 2.496 GHz, 1 mW max power) that is Food and Drug Administration–cleared for migraine and cluster headaches.^2^

The SD covered his scalp, forehead, nose, nasolabial folds, cheeks, lips, and chin. The patient reported that his SD was worsened by mental stress. He said he washed his scalp every 2 days with a zinc pyrithione shampoo. He last used tacrolimus 0.1% ointment with some success 2 months ago.

**Fig 1:**
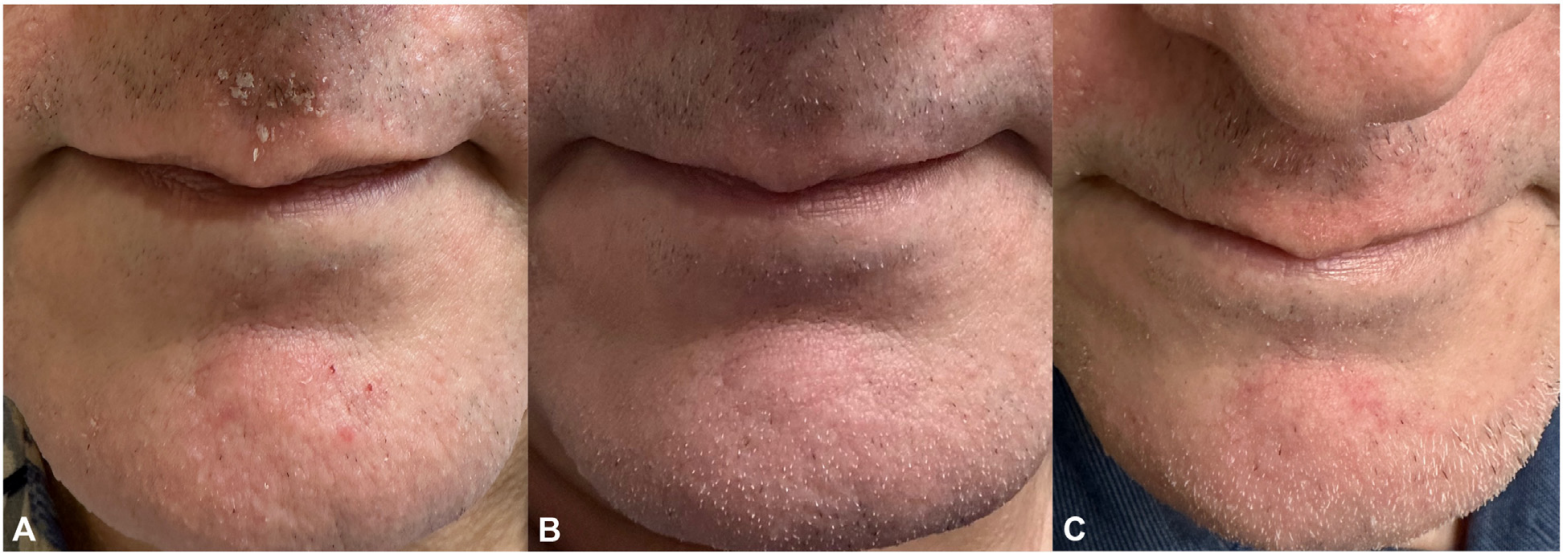
SD on the cutaneous surface of the upper lip and chin. **A,** Baseline pretreatment image with SEDASI full-face moderate score of 23. **B,** Improvement in SD from baseline after 5 days of tVNS treatment with SEDASI full-face mild score of 8. **C,** Mild worsening of SD from best improvement stage after 19 days of tVNS with SEDASI full-face score of 14. *SD*, Seborrheic dermatitis; *SEDASI*, SEborrheic Dermatitis Area and Severity Index; *tVNS*, transcutaneous vagal nerve stimulation.

The patient’s SD was assessed with the SEborrheic Dermatitis Area and Severity Index (SEDASI) (1-14 mild; 15-29 moderate; 30-44 severe; > 45 very severe) that evaluates 4 facial regions.^5^ The scalp SD was not evaluated. Prior to the actual employment of tVNS, his SEDASI score on the face was 23, graded as moderate^5^ (Fig 1, *A*). The tVNS device, which emits electrical impulses, was applied over 1 side of the cervical neck area where the carotid pulse is felt. Energy settings were increased to a point where the device elicited a downturn of the lower lip. The patient reported employing 2 consecutive 2-minute sessions within 1 hour of awakening and before sleeping.

After employing the tVNS for 5 days, he not only experienced a reduction in headache severity and frequency but also noticed a marked improvement in his SD on the face, including the upper lip. His SEDASI dropped to a mild score of 8 (Fig 1, *B*). After continuing the tVNS for 2 additional weeks, he noticed the SD slightly worsen and stabilize, but at an improved state from the pretreatment baseline with a SEDASI mild score of 14 (Fig 1, *C*). After 3 months of continuous tVNS (SEDASI of 14), the patient had a dechallenge of treatments for 2 weeks, and his SD worsened back to beyond baseline status with a SEDASI of 24.

## Discussion

The precise etiopathogenesis remains incompletely elucidated for SD. Current evidence suggests several pathophysiologic mechanisms in the development of SD: hyperactive sebaceous gland secretion with subsequently altered lipid composition, proliferation of *Malassezia* species, and an aberrant host immune response that confers susceptibility to the condition.^6^ In SD, the pathogenic cascade is initiated by *Malassezi*a-derived lipase activity, which catalyzes the hydrolysis of sebum triglycerides, generating free fatty acids and lipid peroxidation products. This metabolic process facilitates further *Malassezia* colonization and triggers an inflammatory cascade. The resultant immune response is characterized by cytokine upregulation, which induces keratinocyte hyperproliferation and altered differentiation, culminating in epidermal barrier dysfunction—the hallmark of SD.^6^ While the inflammation profile is complex, several cytokines have demonstrated robust correlation with SD pathogenesis, notably interleukin (IL)–17, IL-1α, IL-2, and interferon-γ.^6^

Preclinical studies have demonstrated that VNS decreased proinflammatory cytokines such as IL-1β, IL-6, and tumor necrosis factor-α.^4^^,^^7^ Also, VNS was shown to block activation of IL-6 and interferon-γ in patient studies and increased the anti-inflammatory cytokine IL-10 in preclinical studies.^7^ While the complex interplay of various cytokines through the spleen and plasma likely contributes to the multifaceted nature of SD, the improvement in SD may be related to the modulation of interferon-γ with VNS therapy.

Neural adaptation describes the progressive diminution of neuronal response to sustained or repetitive stimulation. There appears to be a mild, temporary adaptation with tVNS for SD (SEDASI of 8 to 14) between 5 and 19 days. Still, the adaptation disappeared as continued tVNS treatments did not further diminish the SD improvement, and the patient stabilized with an SEDASI of 14 over several months.

While no evidence supports VNS as a therapeutic approach for refractory SD, the established association between refractory SD and various neurological disorders provides a theoretical foundation for its efficacy. Moreover, the benefits of VNS in epilepsy, depression, stroke, and Parkinson’s disease suggest that this intervention may offer similar advantages in cases of refractory SD associated with these comorbidities.^2^^,^^8^

Vagal nerve dysregulation and autonomic disbalance have been shown to affect many morbidities, including obesity, diabetes, arthritis, cardiac arrest, acute myocardial infarction, and the aforementioned neurological morbidities.^2^^,^^8^^,^^9^ SD and other inflammatory skin disorders have been associated with these and some autoimmune conditions.^10^ The dysregulation of the vagus nerve could be the conduit that links comorbid conditions to SD and other inflammatory skin conditions.

This treatment is the first reported case of tVNS that has been shown to impact an inflammatory skin disorder. tVNS may be a safe and noninvasive alternative treatment that may modulate the outcomes of other inflammatory skin disorders and their comorbid conditions. tVNS, with its potential treatment in inflammatory skin diseases, like SD, should be more extensively studied.

## Conflicts of interest

None disclosed.
